# A novel anoikis-related gene signature identifies LYPD1 as a novel therapy target for bladder cancer

**DOI:** 10.1038/s41598-024-53272-0

**Published:** 2024-02-08

**Authors:** Zhen Song, Shikai Gui, Shuaiyun Xiao, Xuepeng Rao, Na Cong, Huanhuan Deng, Zhaojun Yu, Tao Zeng

**Affiliations:** 1 Department of Urology, The Second Affiliated Hospital of Nanchang University, Nanchang, 330000 Jiangxi Province China; 2 Nanchang University, Nanchang, 330000 Jiangxi Province China; 3 Department of Neurosurgery, The Second Affiliated Hospital of Nanchang University, Nanchang, 330000 Jiangxi Province China; 4 Ganzhou Medical Emergency Center, Ganzhou, 341000 Jiangxi Province China

**Keywords:** Cancer, Cell biology, Computational biology and bioinformatics, Immunology, Molecular biology

## Abstract

Bladder cancer (BLCA) is a malignant tumor associated with unfavorable outcomes. Studies suggest that anoikis plays a crucial role in tumor progression and cancer cell metastasis. However, its specific role in bladder cancer remains poorly understood. Our objective was to identify anoikis-related genes (ARGs) and subsequently construct a risk model to assess their potential for predicting the prognosis of bladder cancer.The transcriptome data and clinical data of BLCA patients were sourced from The Cancer Genome Atlas and GEO database. We then performed the differential expression analysis to screen differentially expressed ARGs. Subsequently, we conducted non-negative matrix factorization (NMF) clustering analysis to establish molecular subtypes based on the differentially expressed ARGs. The CIBERSORT algorithm was used to estimate the quantification of different cell infiltration in BLCA tumor microenviroment. A prognostic risk model containing 7 ARGs was established using Lasso-Cox regression analysis. The nomogram was built for predicting the survival probability of BLCA patients. To determine the drug sensitivity of each sample from the high- and low-risk groups, the R package “pRRophetic” was performed. Finally, the role of LYPD1 was explored in BLCA cell lines.We identified 90 differential expression ARGs and NMF clustering categorizated the BLCA patientss into two distinct groups (cluster A and B). Patients in cluster A had a better prognosis than those in cluster B. Then, we established a ARGs risk model including CALR, FASN, FOSL1, JUN, LYPD1, MST1R, and SATB1, which was validated in the train and test set. The results suggested overall survival rate was much higher in low risk group than high risk group. The cox regression analysis, ROC curve analysis, and nomogram collectively demonstrated that the risk model served as an independent prognostic factor. The high risk group had a higher level TME scores compared to the low risk group. Furthermore, LYPD1 was low expression in BLCA cells and overexpression of LYPD1 inhibits the prolifearation, migration and invasion.In the current study, we have identified differential expression ARGs and constructed a risk model with the promise for guiding prognostic predictions and provided a therapeutic target for patients with BLCA.

## Introduction

Bladder cancer, a frequently encountered neoplasm within the urinary tract, exhibits a progressively escalating trend in both morbidity and mortality rates over temporal progression, constituting a sustained menace to humans^1^. The enhanced comprehension of the molecular biology and genetics of bladder cancer has engendered a paradigm shift in the diagnostic and therapeutic approaches employed for both incipient and advanced manifestations of this malady^2,3^. Nevertheless, there has been a substantial expansion in therapeutic choices for muscle-invasive and advanced disease. Encompassing immunotherapy featuring checkpoint inhibition, targeted therapeutic interventions, and antibody–drug conjugates^4^.

Anoikis is a widely recognized form of programmed cell death that occurs when cells detach from the appropriate extracellular matrix, disrupting integrin ligation^5^. Integrin ligation plays a crucial role in preserving normal development and tissue balance, whereas the acquisition of resistance to anoikis is essential for promoting cancer invasiveness and facilitating the formation of metastases^6^. Emerging research has demonstrated the significant involvement of anoikis in bladder cancer, where resistance to this process can promote the proliferation of bladder cancer cells through various pathways^7^. Therefore, the identification and prediction of anoikis resistance in patients with bladder cancer hold significant potential as valuable tools for predicting disease progression and improving treatment effectiveness.

In this study, we developed a risk model that integrates genes associated with anoikis based on utilizing data from the TCGA and GEO databases. Subsequently, we proceeded to validate the model and conducted a more extensive investigation into the relationship between these genes and the immune microenvironment, as well as their impact on immune function. Additionally, we assessed the sensitivity of various chemotherapeutic agents using the risk score derived from our analysis. Moreover, we discovered that LYPD1 was down-regulated in BLCA cells, and decreased expression of LYPD1 was demonstrated inhibitory effects on the proliferation and invasiveness of BLCA cells in vitro. Through our investigation, our objective is to provide valuable insights into the prognostic importance of genes related to anoikis and highlight potential therapeutic targets for patients with bladder cancer.

## Materials and methods

### Data acquisition

We obtained the genomic data and the accompanying clinical data of BLCA from the TCGA (https://portal.gdc.cancer.gov/) and GSE13507 cohorts, which is accessible through the GEO (http://www.ncbi.nlm.nih.gov/geo/) database. The data of gene expression, somatic mutation, and corresponding clinical information of BLCA samples from TCGA database. We excluded various batches of sample data from the same patient and ultimately collected a total of 655 samples (consisting of 597 tumor tissue cases and 68 normal tissue cases). Additionally, we obtained 753 Anoikis-related genes (ARGs) from GeneCards database (https://www.genecards.org/). To identify differentially expressed genes (DEGs), we set the thresholds at a FDR (false discovery rate) < 0.05 and |log2Fold Change (FC)|> 1.

### Analysis of prognostic relevance and estimation of copy number variation

We used the R package “limma”, “survival”, and “survminer” to integration of prognostic data and establish relationships between differential gene expression. Then, we get the copy number (genes level) from the UCSC database, and use the copy number variation (CNV) algorithm to calculated the frequencies among ARGs.

### Cluster typing

We cluster typing by non-negative matrix(NMF), the clustering of the data based on ARGs expression was performed using the “ConsensusClusterPlus” R package. For various subgroups of bladder cancer patients, the “Rtsne” R package was used to carry out principal component analysis (PCA), T-distributed stochastic neighbor embedding (t-SNE). Additionally, the Kaplan–Meier survival analysis, immunocorrelation analysis, and KEGG analysis^8–10^ was performed.

### Construction and verification the anoikis-related model nomogram

After performing univariate Cox regression analysis, ARGs associated with prognosis were identified. The Lasso regression analysis and multivariate Cox regression analysis was used to develop a novel prognostic gene signature. The risk score for each sample was determined using the formula equation:

$$riskscore=\sum_{i=1}^{n}(coefi*Xi)$$

We then classified bladder cancer patients into a low-risk group and a high-risk group based on the median risk score obtained from the risk model. The ROC curve, area under the ROC curve (AUC) and the survival curve was used to validate the accuracy and prognostic value of the model.

### Development and valuation of nomogram

Risk score, age, gender, grade and stage were analyzed using Multivariate Cox regression analysis to screen the factors significantly related to survival. Based on these findings, a nomogram was developed to effectively represent the predictive factors. To evaluate the predictive performance of the nomogram, a time-dependent ROC analysis was conducted to predict overall survival. Additionally, decision curve analysis was employed to assess the clinical net benefit of each model in comparison to both all or none strategies. The optimal model was determined based on the highest calculated net benefit.

### Immune analyses

The Estimation of Stromal and Immune cells in Malignant Tumor tissues using Expression data (ESTIMATE) method was utilized to calculate the stromal score, immune score and ESTIMATE score. Furthermore, the abundance of 22 tumor-infiltrating immune cells in BLCA samples was determined using the CIBERSORT algorithm through the R package.

### Evaluation of drug sensitivity

In order to evaluate the risk scores for guiding therapy in BLCA, we employed the “pRRophetic” program to determine the IC50 (half-maximal inhibitory concentration) of various chemotherapeutic drugs. Differences in IC50 values between the two risk groups for commonly used antineoplastic drugs were analyzed using the Wilcoxon test. Moreover, the “ggpubr” package was utilized to generate correlation graphs.

### Total RNA isolation and quantitative RT‑PCR

Total RNA was extracted from SV-HUC-1, 5637, T24, and UM-UC-3 cells using Trizol reagent (TransGen Biotech, Beijing, China). The extracted RNA was then reverse transcribed into cDNA using the TransScript First-Strand cDNA Synthesis SuperMix kit (TransGen Biotech, Beijing, China). RT-PCR experiments were performed using qPCR SYBR Green SuperMix (Servicebio, China), and the reaction was conducted as follows: 95 °C for 30 s, 42 cycles of 95 °C for 15 s and 60 °C for 30 s. The relative gene expressions were normalized using the − 2 − ΔΔCT method, with β-Actin serving as the internal reference gene. The primer sequences used in this study were as follows: LYPD1, F: 5′-GGCAACTTTTTGCGGATTGTT-3′, R: 5′-CGTTCACCGTGCAATTCACA-3′, GAPDH, F: 5′-AGGTGAAGGTCGGAGTCAAC-3′, R: 5′-CGCTCCTGGAAGATGGTGAT-3′.

### Cell culture and transfection

The bladder cancer cell lines 5637, T24, UM-UC-3 and human uroepithelial cell line SV-HUC-1 were purchased from Procell Life Science&Technology Co.,Ltd. Cells were cultured in RPMI-1640, DMEM, MEM or Ham’s F-12 medium(Gibco, USA) and the culture medium used in this process includes 10% fetal bovine serum(Biological Industries, Israel). All cells are both incubated at 37 °C in a humidified atmosphere containing 5% CO2. LYPD1 plasmid and control vector were purchased from Miaolingbio (Miaolingbio, China). Plasmid transfection was carried out using the Lipofectamine 3000 transfection reagent (Thermo Fisher Scientific, USA) in 6-well plates according to the manufacturer's instructions. The overexpression level of LYPD1 was assessed through qPCR analyses.

### Cell counting Kit‑8 assay

Cells were seeded in 96-well plates at a density of 2000 cells per well and incubated for 24 h at 37 °C with 5% CO_2_. After the specified incubation period, each well was treated with 10 μl of CCK8 solution. The absorbance was then measured at 450 nm using a multi-scan spectrophotometer two hours later.

### Colony formation assay

Approximately 500 transfected cells were plated in 6-well plates containing culture medium supplemented with 10% FBS and incubated about 7–10 days. The cell colonies were then fixed with 4% paraformaldehyde for 30 min and subsequently stained with 0.1% crystal violet for another 15 min. Finally, high-definition digital images were captured using a camera and analyzed using ImageJ software.

### Migration and invasion assays

To assess the invasion or migration capability of BLCA cells, a transwell membrane (Nest, 0.8 μm pore size) system with or without Matrigel was employed. A total of 2 × 10^4 cells were added into the upper chamber, which was filled with 200 μl of FBS-deficient medium. Simultaneously, 600 μL of medium containing 20% FBS was added to the lower chamber. Following incubation for 24 h, 48 h at 37 °C, the chambers were washed with PBS and then fixed with 4% paraformaldehyde for approximately 30 min. The cells on the upper side of the membrane were gently scraped off using a cotton swab and subsequently stained with crystal violet at room temperature for about 30 min. After washing the membranes with PBS, they were allowed to dry and then photographed.

### Statistical analysis

Statistical diagrams based on the results were generated using R software (version 4.0.2) and GraphPad Prism 8. Student’s t-test and Wilcoxon signed-rank test were used to explore the differences between these subgroups. Generally, a *p*-value less than 0.05 was considered statistically significant. It is worth noting that all experiments were repeated three times to ensure reliability and consistency of the results.

## Results

### Identification of differentially expressed ARGs and overview of genetic changes in BLCA

**Figure 1:**
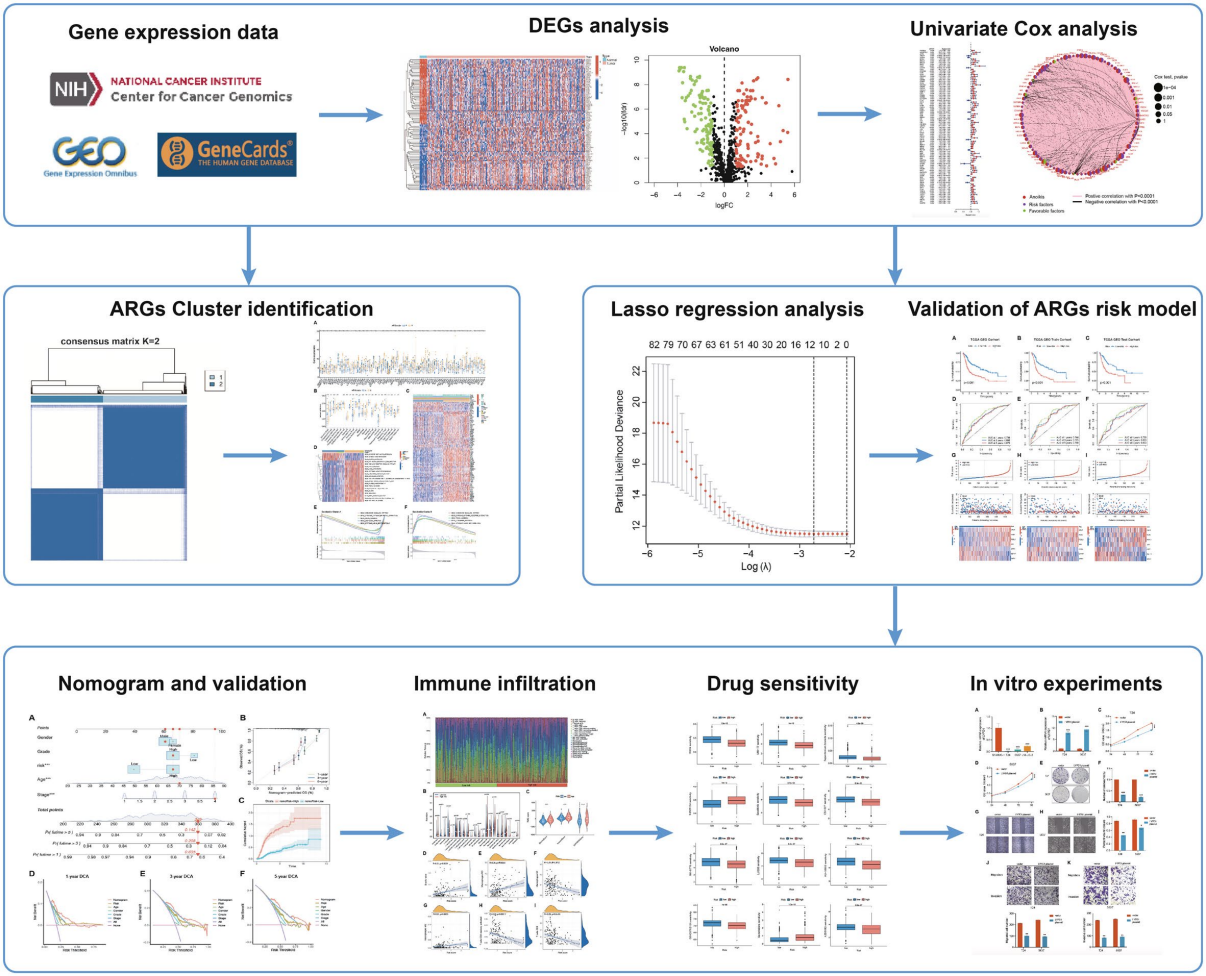
The workflow of this study.

**Figure 2:**
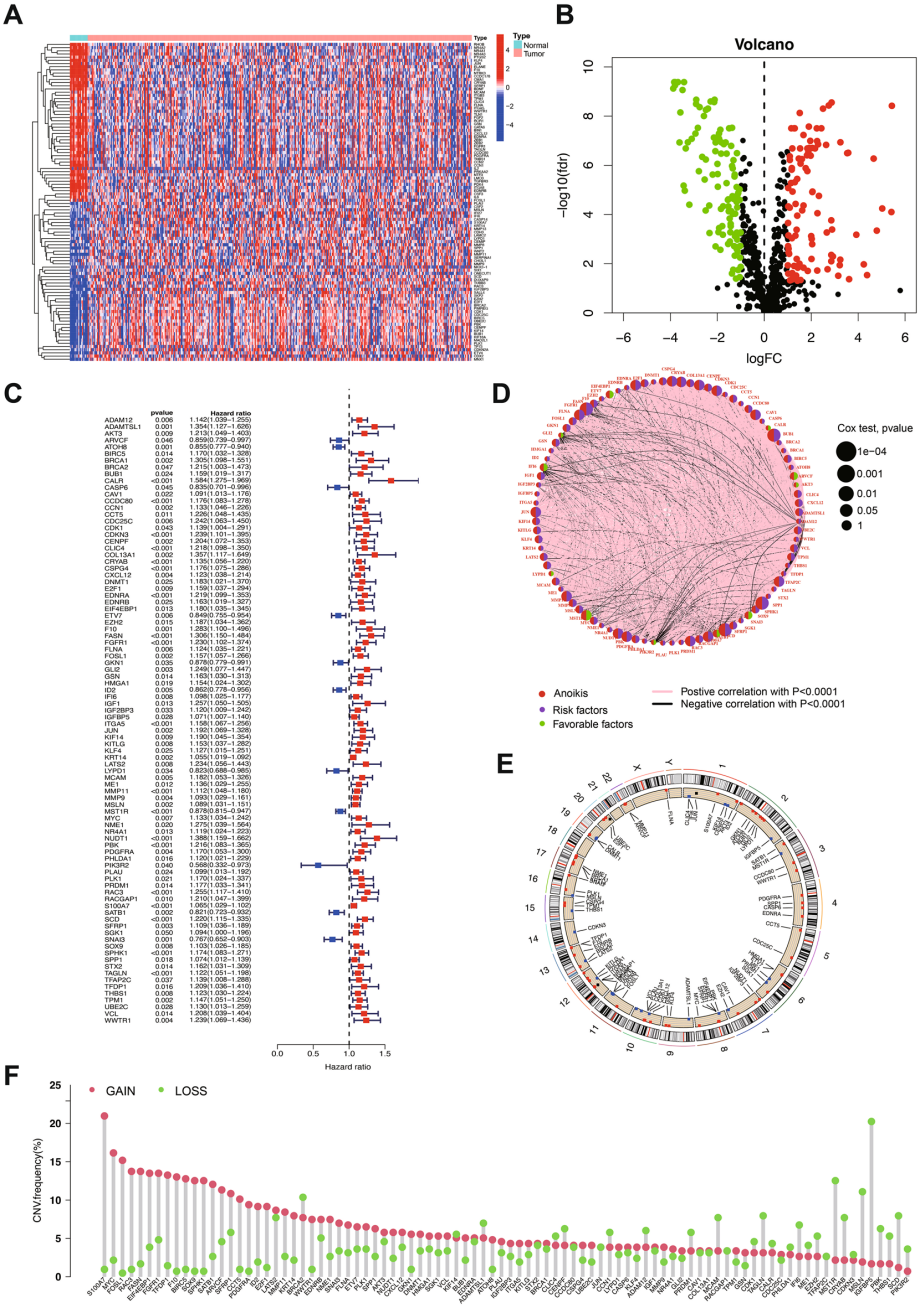
ARGs identification and expression in bladder cancer. (**A**) Heatmap of differentially expressed ARGs. (**B**) Volcano plot indicates ARGs. (**C**) Univariate Cox analysis demonstrated the correlation between anoikis-associated genes and prognosis. (**D**) A circle plot illustrates the correlations among these ARGs. (**E**) An analysis of ARGs with CNVs located at different chromosomal locations. (**F**) Copy number variations (CNVs) of ARGs in patients with BLCA.

The flow chart of whole procedure is shown in (Fig. 1). Initially, we retrieved a total of 753 Anoikis-Related Genes (ARGs) from the GeneCard database (Table S1). Following this, we acquired RNA-seq data from TCGA-BLCA dataset and conducted a differential expression analysis. After merging this data with the GSE13507 dataset, we identified a total of 190 differentially expressed ARGs (Fig. 2A,B). Then, we integrated clinical information of 655 BLCA patients from TCGA-BLCA database and GSE13507 dataset, and used their survival information to conduct univariate Cox regression analysis, leading to the identification of 90 prognosis-associated ARGs (Fig. 2C,D). Copy Number Variations (CNVs) frequently occur in diverse tumors and contributing to the initiation and progression of cancers^11^. The frequency of copy number variants (CNVs) in prognosis-associated ARGs was determined, and our analysis revealed chromosomal changes involving these genes (Fig. 2E,F).

### The molecular subtypes of anoikis-associated genes

**Figure 3:**
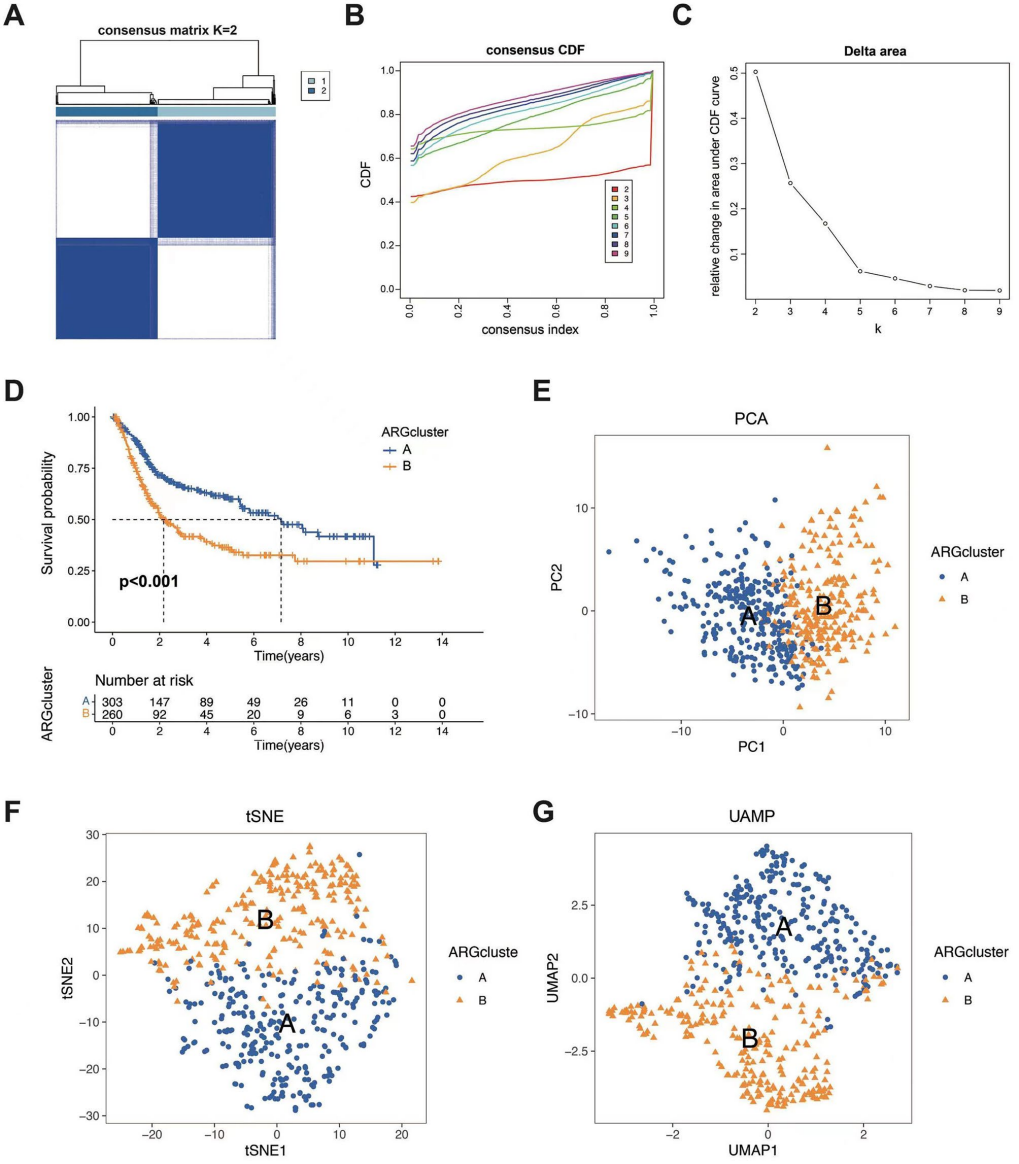
Clustering of ARGs. (**A**) The consensus clustering in BLCA samples with k = 2. (**B**–**C**) consensus clustering CDF for k = 2–9. (**D**) Kaplan–Meier (KM) survival analysis of two subgroups. (**E**–**G**) The PCA, tSNE and UAMP analysis of different subtypes.

The NMF method was utilized to cluster the combined transcriptome data from both TCGA and GEO datasets. The assessment of stability and clustering performance relied on cophenetic and RSS measures. The most optimal clustering outcome was achieved with K = 2, indicating successful classification of samples into two distinct groups, denoted as cluster A and cluster B (Fig. 3A–C). Notably, significant differences between these two groups were observed based on clinical data. Particularly, Cluster B is associated with a poorer prognosis compared to Cluster A (Fig. 3D). Additionally, the accuracy of the NMF method is demonstrated by PCA, tSNE, and UMAP analyses (Fig. 3E–G).

### Identification the biological characteristics of ARGs subtypes

**Figure 4:**
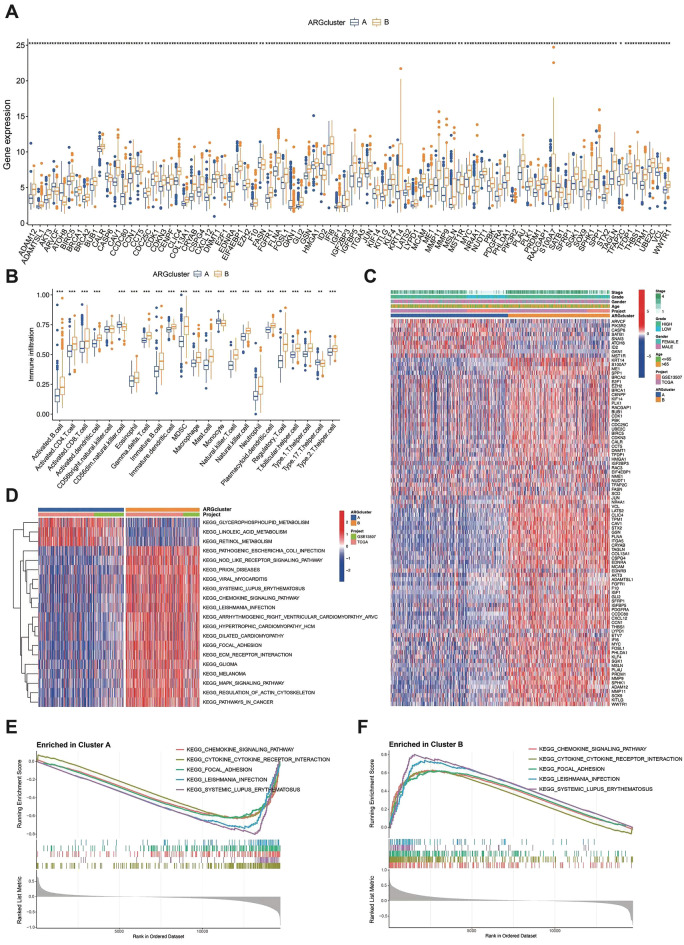
The functional analysis of ARGs. (**A**) The 84 differential expressed genes between the ARGs cluster A and B. (**B**) Boxplots depicting the 28 immune signature ssGSEA scores of the ARGs cluster A and B. (**C**) Difference distribution of clinicopathological features and ARGs expression among the two subtypes. (**D**) The gene set variation analysis (GSVA) of the differences in KEGG pathways within subgroups A and B. (**E**–**F**) GSEA between clusters A and B.

Next, we identify 84 differential expressed genes between the ARGs cluster A and B (Fig. 4A), and the ARGs cluster B patterns were also linked to stages and grade (Fig. 4C). Furthermore, we used the CIBERSORT algorithm to estimate the quantification of different cell infiltration in BLCA sample, and the proportions of immune cell and stromal cell infiltration ratios in C1 and C2 also exhibit significant distinctions (Fig. 4B). Specifically, cluster B had higher levels of infiltration of activated memory CD4+T cells, CD8+T cells, activated NK cells, M1 macrophages, and dendritic cells, while cluster A is very abundant in Monocyte cell. Pathway analysis was performed on the different subtypes to identify their corresponding characteristics. The GSVA results indicated enrichment of pathways such as MAPK signaling, ECM-receptor interaction, and pathogenic Escherichia coli infection in cluster B. In contrast, cluster A was enriched in pathways related to retinol metabolism, linoleic acid metabolism, and glycerophospholipid metabolism (Fig. 4D). Notably, cluster B demonstrated enrichment in pathways like chemokine signaling, cytokine receptor interaction, focal adhesion, leishmania infection, and systemic lupus erythematosus (Fig. 4E,F).

### Creating a risk assessment model

**Figure 5:**
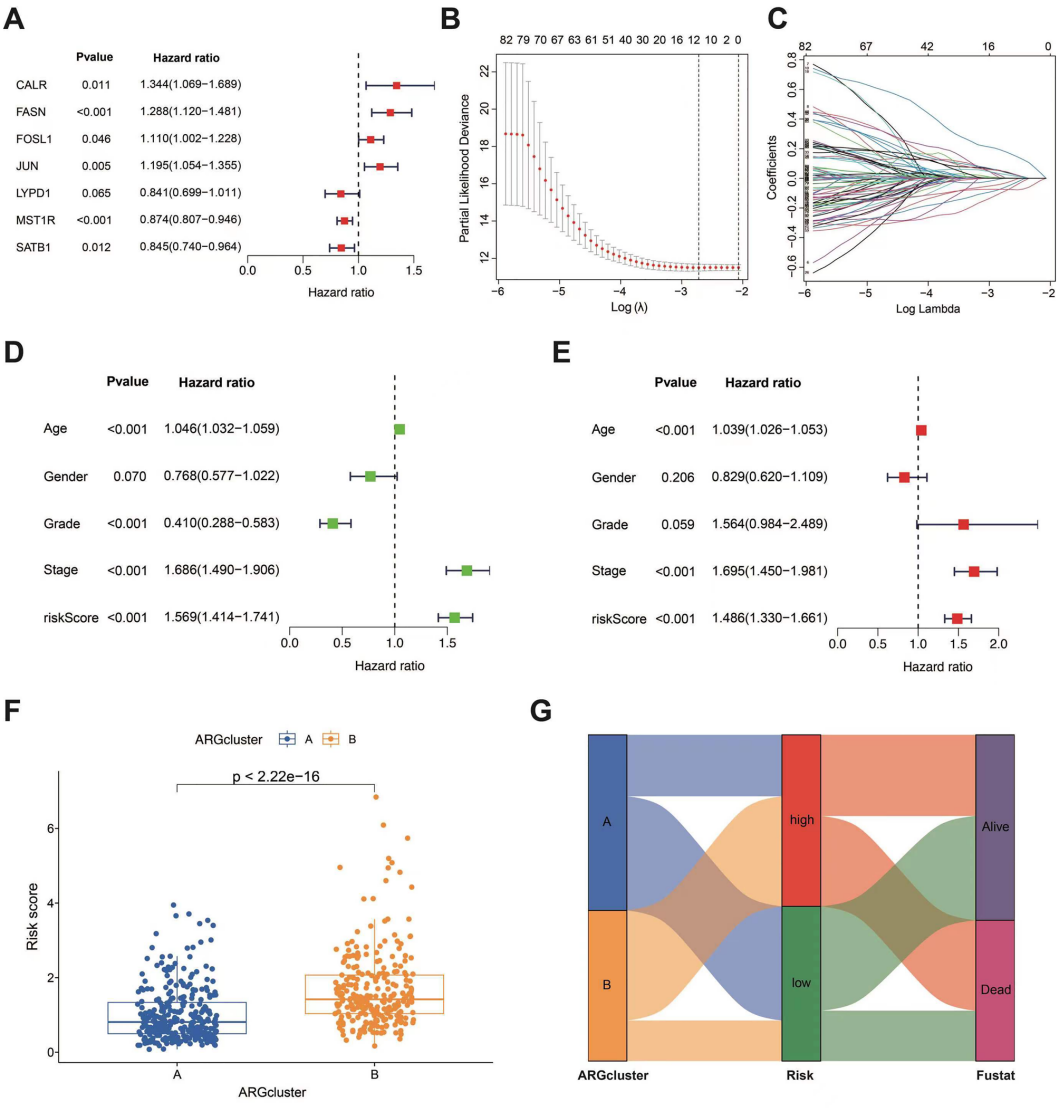
The risk model based on ARGs. (**A**) Univariate Cox regression analysis was performed for 90 ARGs. (**B**) Coefficient of the 7 ARGs. (**C**) Curve of error rate tenfold cross-validation. (**D**-**E**) Uni- and multi-Cox analyses of clinical features. (**F**) Comparison of risk scores of the 2 subgroups. (**G**) Sankey diagram of interrelationship between three subtypes and high and low risks.

Based on the 90 prognosis-associated ARGs that identified through univariate Cox regression analyses, we constructed a ARGs prognosis risk model by LASSO Cox regression analysis (Fig. 5A–C). The risk score for each BLCA patient was computed using the following formula: risk score = CALR × (0.405279308953595) + FASN × (0.258102908642283) + FOSL1 × (0.13711645977668) + JUN × (0.180089629387815) + LYPD1 × (− 0.297294747687542) + MST1R × (− 0.158806008985081) + SATB1 × (− 0.258123805382691) (Table S2). Univariate Cox regression analyses indicated that age, grade, stage and risk score were significantly correlated with prognosis in patients with BLCA from TCGA-BLCA database and GSE13507 database (Fig. 5D). Moreover, the age, stage and risk score was found to be a distinct prognostic marker in the multivariate Cox regression analysis (Fig. 5E). The cluster B had a high risk score than cluster A (Fig. 5F). Additionally, the Sankey diagram illustrates the distribution of BLCA patients across ARG subtypes, risk scores, and prognostic outcomes, as shown in Fig. 5G.

### Validation of ARGs signature

**Figure 6:**
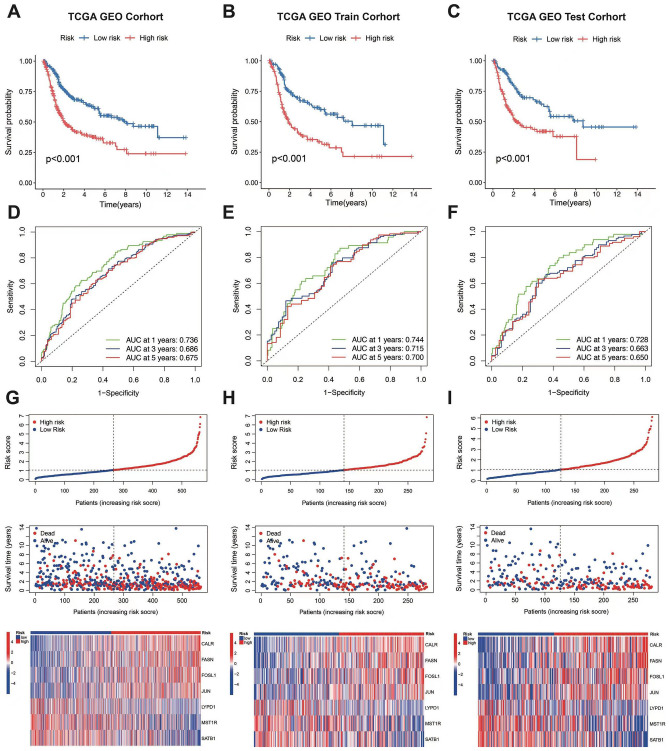
Validation of ARGs signature. (**A**–**C**) The Kaplan–Meier survival curve of the OS rate in different subgroups of patients with BLCA. (**D**–**F**) 1-, 3-, and 5-year ROC curves of the all,training and testing set. (**G**–**I**) Survival plots and The heat map of 7 ARGs expression in each subgroups.

The BLCA patients were categorized into low and high risk groups based on their median risk score (1.053). KM survival analysis demonstrated that the low risk group exhibited a more favorable prognosis (Fig. 6A). The BLCA patients was divided randomly into train cohort and test cohort, KM survival analysis was conducted on both corhorts to verify the conclusions mentioned earlier. The results obtained from both the train cohort and the test cohort were consistent with the previous analysis (Fig. 6B,C). The ROC curves for 1-, 3-, and 5-year OS were 0.736, 0.686, and 0.675, respectively(Fig. 6D). These results were further verified in train and test cohorts (Fig. 6E,F). In a word, these findings suggest that the established prognostic signature has a high predictive accuracy at different time points, indicating its effectiveness in predicting patient survival over specific durations. Furthermore, the prognostic curve and scatter plot depicted the risk score and survival status of each patient (Fig. 6G–I).

### Constructing a nomogram and calibration curves

**Figure 7:**
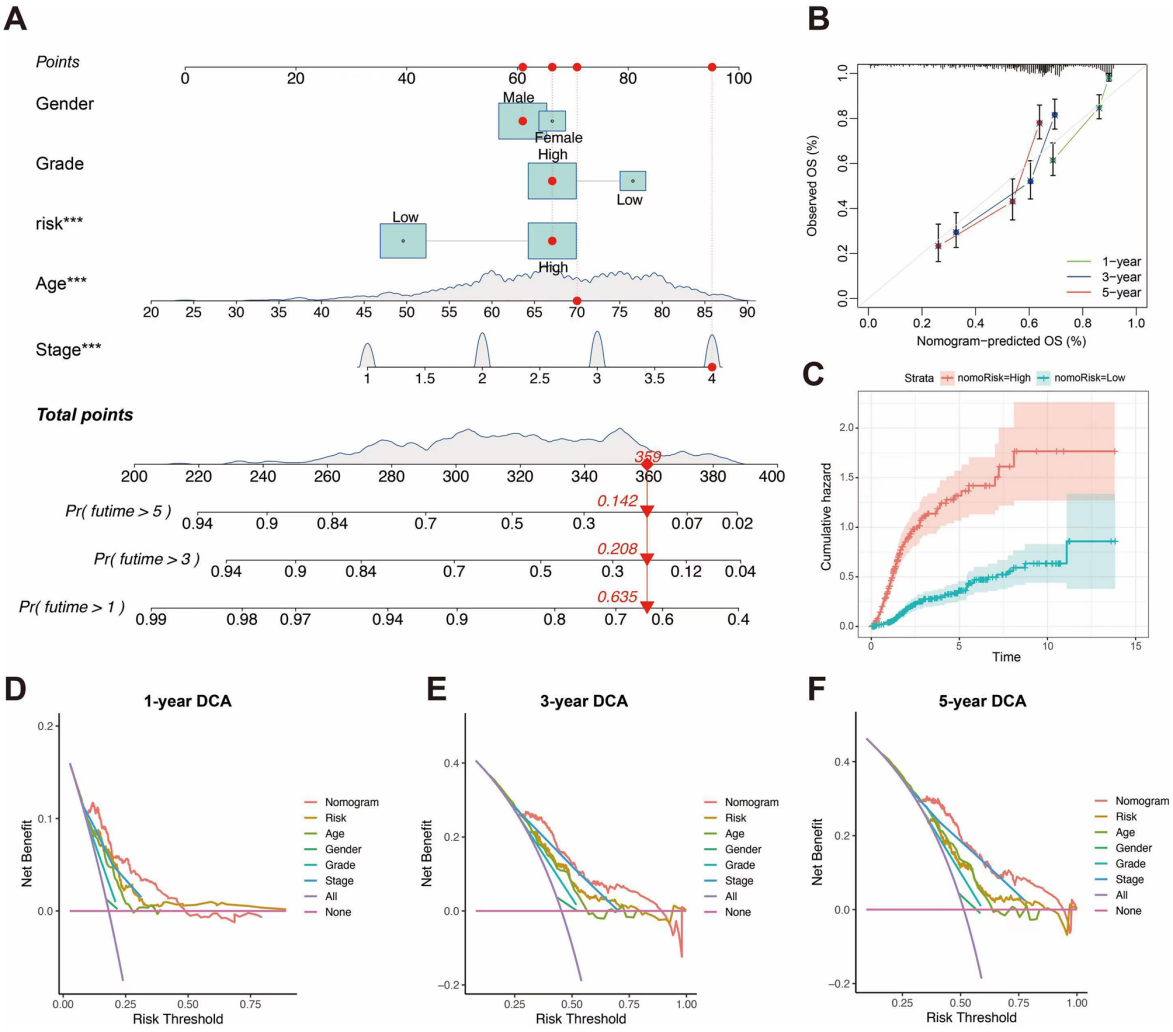
Nomogram to predict survival probability of bladder cancer patients. (**A**) Predictive nomogram for survival over 1-, 3-, and 5-years based on risk groupings and clinical characteristics. ***p < 0.001. (**B**) Comparison of actual and predicted outcomes at 1-, 3-, and 5-years based on calibration curves. (**C**) The plot of CIR after risk stratification of the nomogram. (**D**–**F**) The decision curve analysis (DCA) of risk scores and clinical features at 1-, 3-, and 5- years.

To explore an enhanced quantitative approach for predicting the prognosis of BLCA patients, we developed a nomogram that integrates age, gender, grade, stage and risk scores (Fig. 7A). Additionally, the calibration curves for 3- and 5-year OS demonstrated strong reliability between the prognostic predictions generated by the nomogram and the actual observed outcomes (Fig. 7B), and the results of the Decision Curve Analysis (DCA) analysis further indicated that the nomogram exhibited a superior level of clinical utility compared to traditional clinical indicators at 1, 3, and 5 years (Fig. 7C–F).

### Tumor microenvironment cell infiltration and investigating potential clinical interventions

**Figure 8:**
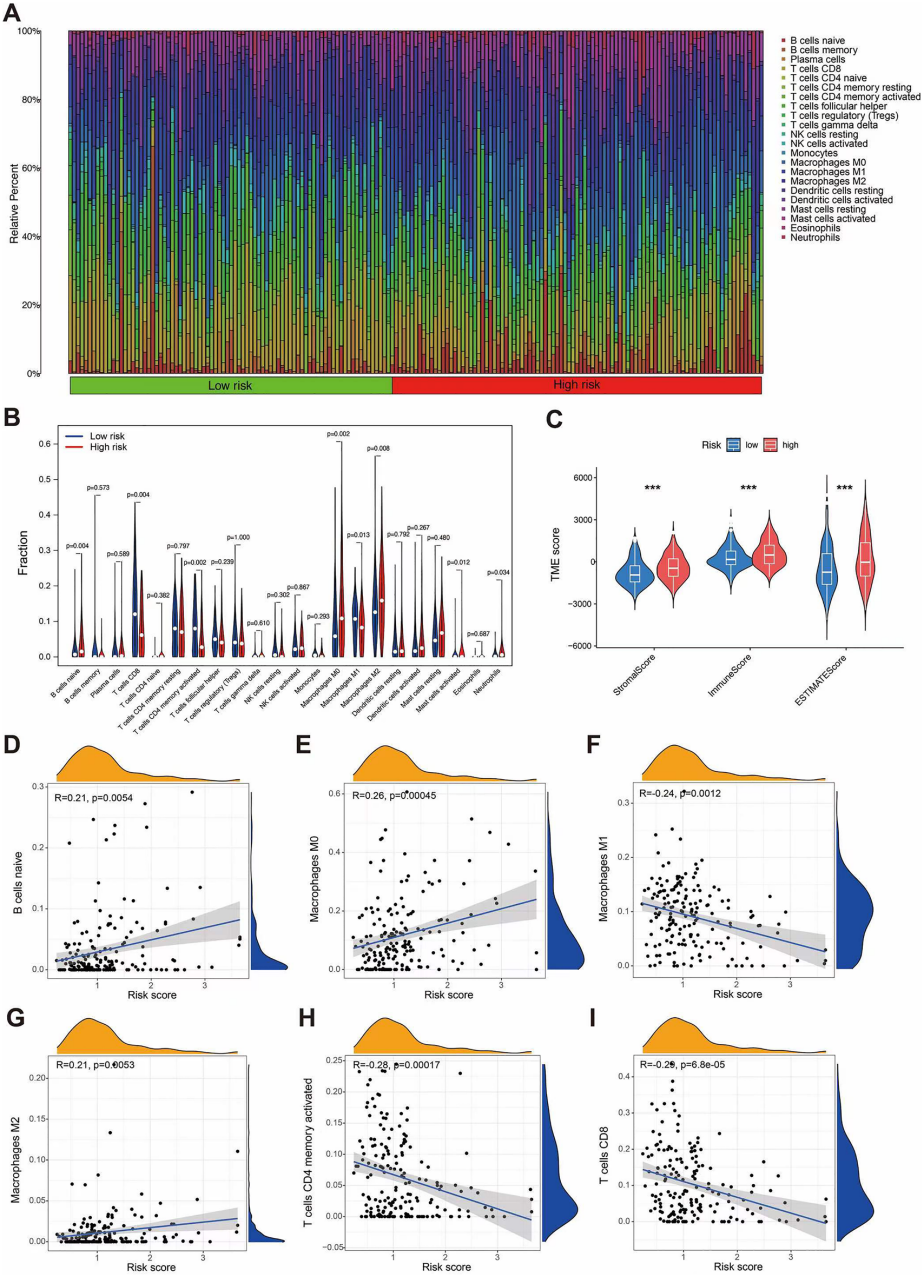
Tumor microenvironment cell infiltration. (**A**–**B**) An illustration of the fractions of 22 types of infiltrating immune cells for low and high risk patients. (**C**) TME scores between the low and high risk patients. (**D**–**I**) The infiltrating levels of different immune cells in the high and low risk groups.

**Figure 9:**
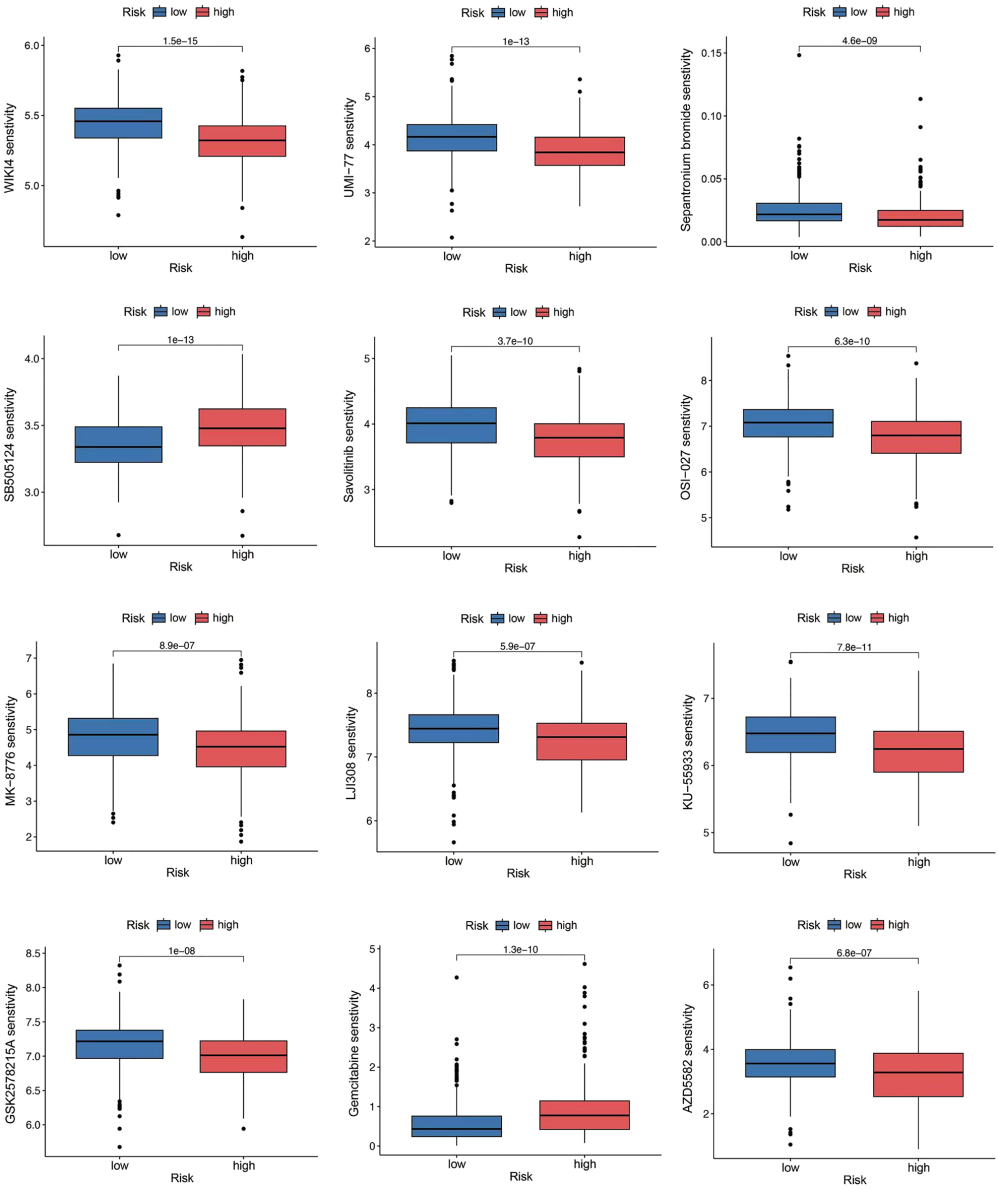
Drug sensitivity analysis in high and low risk groups.

Given the significant impact of the tumor immune microenvironment on therapeutic outcomes and patient prognosis in malignant tumors, we conducted an analysis to examine the prevalence of distinct immune cell types within both low and high risk subgroups using the TCGA and GSE13507 datasets (Fig. 8A). Specifically, we observed a notable increase in the abundance of Macrophages M0, Macrophages M2, Dendritic cells resting, and Mast cells activated in high risk subgroup (Fig. 8B). The ESTIMATE analysis demonstrated that patients within the high risk group displayed significantly higher stromal, immune and ESTIMATE scores (Fig. 8C). Notably, the risk score was found to postitive correlation with B cells naive, Macrophages M0, Macrophages M2 (Fig. 8D,E,G), while displaying a negative correlation with Macrophages M1, T cells CD4 memory activated, T cells CD8 (Fig. 8F,H,I). These result suggested that higher risk score was positive correlation with a poor prognosis in BLCA patients. The evaluation of drug susceptibility using the “pRophetic” R package and results revealed that the low risk group display higher sensitivity to Axitinib, Cyclophosphamide, Temozolomide, epantronium bromide, and Savolitinib, while the high risk group exhibit greater sensitivity to Uprosertib, Trametinib, Selumetinib, Palbociclib, Oxaliplatin, and Gemcitabine (Fig. 9). This finding offers valuable guidance for tailoring medication strategies for patients with bladder cancer.

### Overexpression of LYPD1 inhibited proliferation, metastasis and invasion in BLCA cells

**Figure 10:**
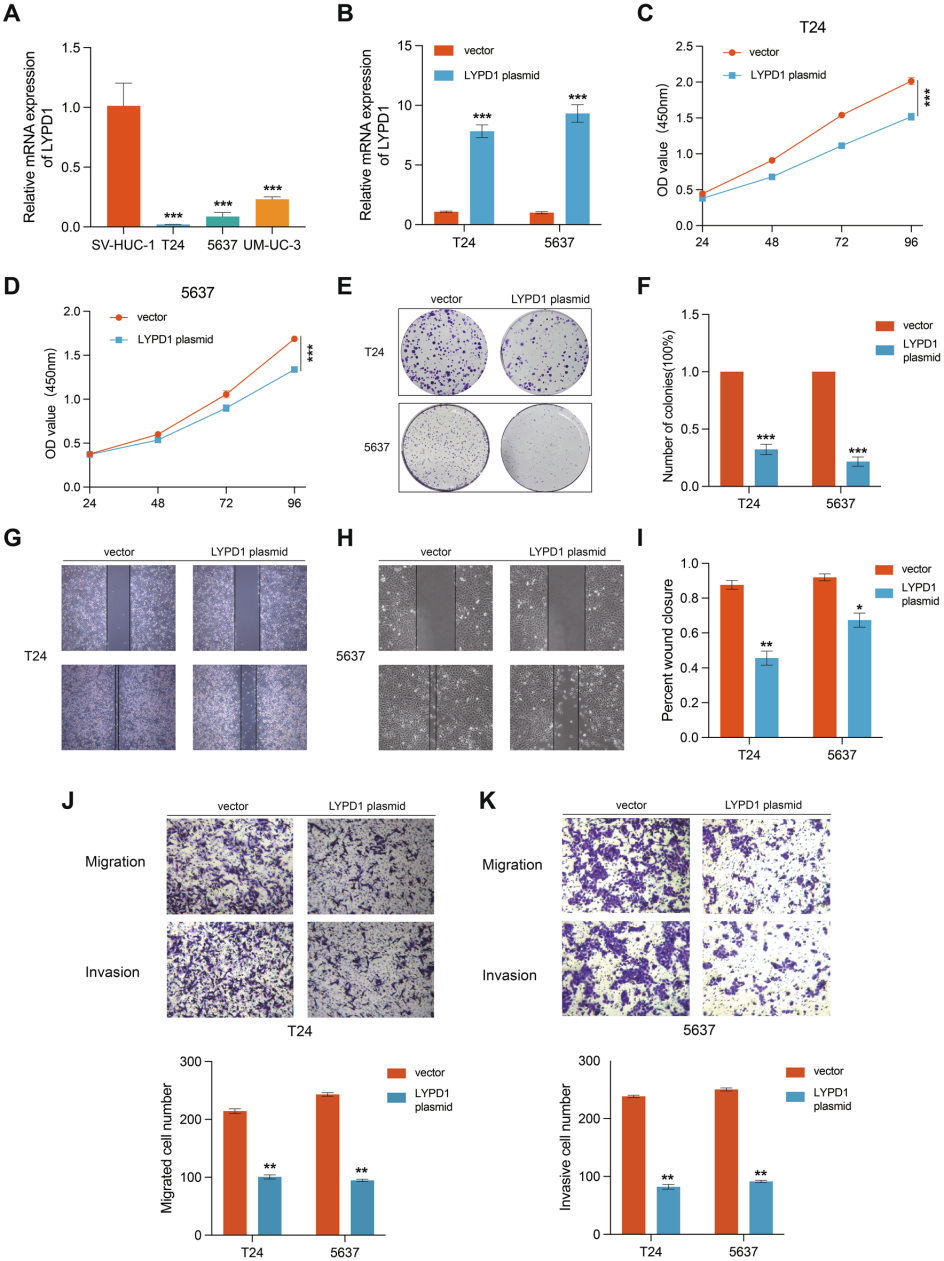
Overexpression of LYPD1 inhibited proliferation, metastasis and invasion in BLCA cells. (**A**) Relative protein expression of LYPD1 in normal bladder cell SV-HUC1 and four BLCA cell lines. (**B**) RT-qPCR and western blot analysis of LYPD1 expression level in the BLCA cells transfected with vector and LYPD1 plasmid. (**C**–**D**) Cell viability was determined by CCK8 assay in the BLCA cell lines transfected with vector and LYPD1 plasmid. (**E**–**F**) Colony formation assay of the LYPD1 overexpression cells. (**G**–**I**) Representative data from wound healing migration assays performed with the LYPD1 overexpression cells. (**J**–**K**) Representative data from transwell migration and matrigel invasion assays performed with the LYPD1 overexpression cells.

We found the mRNA level of LYPD1 was significant downregulation in BLCA cell lines compared to SV-HUC-1 (Fig. 10A). Consequently, we proceeded to investigate its functional role in BLCA cells. To achieve this, we upregulated the LYPD1 levels in T24 and 5637 cells (Fig. 10B), and then employed wound healing and transwell assays to assess changes in the invasion and migration abilities of BLCA cells. The findings demonstrated that LYPD1 overexpression significantly reduced the growth and proliferation in both T24 and 5637 cells (Fig. 10C–F). Additionally, the wound healing and transwell assays revealed that LYPD1 overexpression inhibited the migration and invasion proliferation of BLCA cells (Fig. 10G–K).

## Disscussion

Bladder cancer is a profoundly aggressive urinary tumor^12^. Growing evidence underscores the significance of the extracellular matrix (ECM) as a pivotal component of the tumor microenvironment (TME). Several proteins that regulate the ECM have been linked to the development and unfavorable prognosis of BLCA^13^. Anoikis, which is a specific form of apoptotic cell death, can be triggered when cells lose their interaction with the neighboring extracellular matrix (ECM)^14^. Hence, our study aims to develop a model based on Anoikis-Related Genes (ARGs) to predict the prognosis of bladder cancer patients and investigate potential therapeutic strategies.

Bioinformatics analysis has contributed significantly to the identification of a growing number of molecules implicated in the progression of BLCA^15^. In this study, we employed a combination of Pearson's correlation, differential expression analysis and univariate Cox regression analysis to identify differentially expressed ARGs associated with prognosis. Furthermore, we classified BLCA patients into two distinct subgroups (Cluster A and Cluster B) and cluster A had a high survival rate than cluster B. Approximately all of these ARGs exhibited significantly higher expression levels in cluster B. Intriguingly, our analysis of immune cell infiltration using ssGSEA revealed substantially higher percentages of immune cells in cluster B compared to cluster A. Among these immune cells, macrophages are a significant component of the leukocyte infiltrate in the tumor microenvironment, consisting of M1 and M2 macrophages^16^. Accumulating evidences show that macrophages can promote the tumor proliferation, invasion, and metastasis^17^. The increased immune cell infiltration in cluster B likely contributes to the poorer prognosis, which is consistent with previous results.

We established a prognostic model using LASSO Cox regression analysis, incorporating seven crucial anoikis-related genes (CALR, FASN, FOSL1, JUN, LYPD1, MST1R, and SATB1). Calreticulin (CALR) is an endoplasmic reticulum (ER)-resident protein involved in a spectrum of cellular processes^18–21^. The exposure of CALR on the cell surface can trigger an antitumor immune response in bladder cancer^22^. In bladder cancer, FASN (Fatty acid synthase) can acts as a central regulator of aerobic glycolysis, contributing to the metabolic switch in cancer and promoting tumor cell proliferation^23–25^. The FOSL1 protein is commonly regarded as a subunit of the transcriptional complex AP1, and it plays a critical role in cell differentiation, response to environmental stresses, and tumorigenesis^26,27^. c-Jun N-terminal Kinases (JNKs) have been identified as key disease drivers in a number of pathophysiological settings and central oncogenic signaling nodes in various cancers^28^. The elevated c-Jun activity is positively correlated with the clinical tumor grade in bladder cancer^29–31^. Extensive in vitro and in vivo evidence has demonstrated the significance of MSP-RON(MST1R) signaling in facilitating the invasive growth of various cancer types^32,33^. Several studies suggested that the clinical significance of SATB1 as a potential biomarker for predicting aggressive behavior and poor prognosis in bladder cancer patients^34–36^. LYPD1 is identified as one of the most significant model genes in our study and belongs to the Lynx family of neurotransmitter receptor-binding proteins^37,38^. In ovarian cancer, LYPD1 has been reported to participate in the regulation of ovarian cancer and can function as a novel prognostic marker^39^. Overexpression of LYPD1 causes poor prognosis in hepatocellular adenocarcinoma^40^. An increasing number of studies have demonstrated that LYPD1 plays an important role in tumors, and it has not been reported in bladder cancer. Therefore, we explore the specific function of LYPD1 in BLCA. Our results indicated that the mRNA of LYPD1 was downregulated in BLCA cell lines, and the growth, proliferation, migration and invasion were inhibited with overexpression of LYPD1 in BLCA cells.

Bladder cancer patients were classified into low and high risk group according to the median risk scores. We conducted an analysis of the association between the low and high risk score groups generated by the model and their respective clinicopathological characteristics. Subsequently, multivariate Cox analysis was performed, revealing that the prognostic risk signature demonstrated its independence as a prognostic factor in BLCA patients, and its predictive accuracy was confirmed through internal and external validation. Moreover, we developed a nomogram model by integrating select clinicopathological variables with the risk scores, and this model exhibited robust predictive capability for prognosis. The calibration plots indicated that the nomogram exhibited a good performance for predicting overall survival. The DCA curve further suggests that the nomogram constructed using the seven anoikis-related gene signature can provide benefits to BLCA patients over 1, 3, and 5 years.

Tumor microenvironment (TME) has emerged as a focal point of research due to its role as an inherent oncogenic mechanism and involvement in epigenetic modifications^41^. The interplay among stromal cells, immune cells, and tumor cells constitutes a crucial factor in tumor growth and progression^42^. Through an analysis of the proportions of 22 types of immune cells in both low and high risk groups, we observed a significant upregulation in the infiltration levels of Macrophages M0, Macrophages M2, and activated mast cells in the high-risk group, which was associated with a poorer prognosis. Higher proportion levels of T cells CD8, T cells CD4 memory activated and Macrophages M1 were significantly upregulated in the low risk group. The results of the immune cell infiltration analysis also indicated that the low risk group had a more favorable prognosis compared to the high-risk group. Existing studies also support this claim, with some research indicating a high correlation between the level of immune infiltration and the efficacy of immunotherapy^43–45^. The drug sensitivity analysis was conducted using the 'pRRophetic' package, offers valuable insights into the clinical medication strategies for BLCA patients. These findings have great significance for the clinical treatment of BLCA in clinical practice.

## Conclusion

In summary, we constructed a novel prognostic risk model based on 7 ARGs, which exhibits strong predictive capabilities for prognosis in BLCA patients. Moreover, our findings indicated that increased LYPD1 expression led to the inhibition of BLCA cell proliferation, migration, and invasion. These results underscore the potential of LYPD1 as both a promising therapeutic target and a valuable prognostic indicator for BLCA patients.

### Supplementary Information

Supplementary Information 1.

Supplementary Table S1.

Supplementary Table S2.

## Data Availability

Publicly available datasets were analyzed in this study. This data can be found below: TCGA, https://www.cancer.gov/; GEO, https://www.ncbi.nlm.nih.gov/geo/; GeneCards, https://www.genecards.org/.
