# Supplementary material for: A novel anoikis-related gene signature identifies LYPD1 as a novel therapy target for bladder cancer
Source: Sci Rep. 2024 Feb 8;14:3198. doi: 10.1038/s41598-024-53272-0 (PMC10853254; doi:10.1038/s41598-024-53272-0)
Supplement: Supplementary file 1 — Supplementary Information 1. [file 41598_2024_53272_MOESM1_ESM.docx]

Supplementary Materials:

Table S1: anoikis-related genes from GeneCards;

Table S2: Coefficients based on a multivariate Cox regression analysis.
